# Agentic and communal narcissism in predicting different types of lies in romantic relationships

**DOI:** 10.3389/fpsyg.2023.1146732

**Published:** 2023-07-03

**Authors:** Nico Harhoff, Nina Reinhardt, Marc-André Reinhard, Michael Mayer

**Affiliations:** Department of Psychology, University of Kassel, Kassel, Germany

**Keywords:** grandiose narcissism, agency-communion model of narcissism, self-centered lies, other-oriented lies, romantic relationships

## Abstract

Several studies have investigated a potential positive association between agentic narcissism and general dishonesty, revealing both supportive and contradicting evidence. Few have focused on dishonesty within romantic relationships, a phenomenon that occurs in almost all partnerships. With the present research, we first aimed to extend existing literature on narcissism by including its two complementary facets (i.e., agentic and communal narcissism). Second, we aimed to improve the understanding of narcissists’ lying behavior in the context of partnerships by distinguishing between two different types of lies (i.e., self-centered and other-oriented lies). We hypothesized that both, people higher in agentic and communal narcissism, will report increased dishonesty toward their romantic partners (Hypothesis 1). Given the self-benefit function of self-centered lies and given that agentic narcissists aim to fulfill their relationship-based motives by agentic means, we predict agentic narcissism (compared with communal narcissism) will be a stronger predictor for self-centered lies (Hypothesis 1a). Given the other-benefiting function of other-oriented lies and given that communal narcissists aim to fulfill their motive of self-enhancement by communal means, we predict that communal narcissism (compared with agentic narcissism) will be a stronger predictor for other-oriented lies (Hypothesis 1b). In two preregistered online studies (*N* = 298: *N* = 256) we showed that people higher in agentic narcissism believed to be good liars, but this does not lead to higher self-reported frequencies of other-oriented and self-centered lies within romantic relationships historically; communal narcissism was also not related to self-reported deception. Limitations and directions for future research are discussed.

## 1. Introduction

Even though increased deception between romantic partners is associated with lower relationship satisfaction and conflict avoidance (Peterson, 1996), almost all people periodically lie to their intimate partners (DePaulo and Kashy, 1998; Guthrie and Kunkel, 2013). Cole (2001) identified low commitment and higher levels of avoidant and anxious attachment styles as important predictors for increased deception in romantic partnerships. Recently, Reinhardt and Reinhard (2023) identified the Honesty-Humility factor (from the HEXACO model of personality; Ashton and Lee, 2007) as a key predictor for relationship-based dishonesty.

Not surprisingly, studies have consistently shown agentic narcissism (which is the most researched form of narcissism) to be negatively linked to relationship commitment, indicating that agentic narcissists are less invested in continuing their relationships over the long-term (Campbell et al., 2002; Campbell and Foster, 2002; Foster et al., 2006; Zeigler-Hill et al., 2020). In this vein, past research showed that agentic narcissists mainly strive to fulfill their egocentric goals, also within their partnership. For example, according to narcissists, an ideal partner should be of high status and virtually perfect, so that they can in turn increase their own status and self-esteem through received admiration as well as identification with the partner (Campbell, 1999; Zeigler-Hill et al., 2019). Then, in the relationship with a partner who meets their requirements, narcissists are consistently on the lookout for potentially better ones, as indicated by increased attention to other potential dating partners, more frequent flirting behavior, higher infidelity, and a stronger game-playing love style (Campbell et al., 2002; Campbell and Foster, 2002). With the present work, we aim to gain a deeper understanding of whether narcissism is also a valid predictor for dishonesty within romantic relationships.

Regarding the overall construct of narcissism, it should be noted that we focus exclusively on grandiose narcissism, which in personality research is commonly considered distinct from vulnerable narcissism (Miller et al., 2011, 2017). We thereby provide one of the first studies to capture both, the agentic and the communal facet of grandiose narcissism, as recently proposed by Gebauer et al. (2012) with the introduction of the agency-communion model of narcissism.

Although grandiose narcissists all have the core self-motives of grandiosity, esteem, entitlement, and power, agentic narcissists satisfy these motives through agentic means (“I am the most intelligent person”), and communal narcissists satisfy these motives through communal means (“I am the most helpful person”; Gebauer et al., 2012). Thus, in contrast to agentic narcissism, the communal facet shows positive associations with agreeableness (Gebauer et al., 2012; Rogoza and Fatfouta, 2019), self-reported altruism (Yang et al., 2018), prosociality (Nehrlich et al., 2019), and trustworthiness (Kwiatkowska et al., 2019). However, when objective assessments are considered, these self-attributions do not hold up, instead showing average scores with respect to prosocial actions (Nehrlich et al., 2019; Rogoza et al., 2023) and knowledge in communal domains (Gebauer et al., 2012).

Agentic narcissism has been repeatedly examined in relation to deceptive behaviors, yielding partly contradictory results: some studies demonstrate positive associations between agentic narcissism and lying in academic (Brunell et al., 2011; Azizli et al., 2016) and professional contexts (O’Reilly and Doerr, 2020), whereas other studies reported no such associations (Baughman et al., 2014; Forsyth et al., 2021). Similarly, there exists supportive (Jonason et al., 2014; Zvi and Elaad, 2018; Forsyth et al., 2021) and contradicting evidence (Azizli et al., 2016) for a general tendency to lie more often as a person higher in agentic narcissism.

Two additional studies have shown that in a hypothetical mating scenario involving a secret meeting with an ex-partner, individuals with higher levels of agentic narcissism showed a higher tendency to lie to their current partner while experiencing lower levels of negative affect than less narcissistic individuals (Azizli et al., 2016; Forsyth et al., 2021). By contrast, Baughman et al. (2014) did not find this significant association using the same scenario.

Studies that examined the underlying factors of lying behavior unambiguously support the assumption that agentic narcissists have an inherent lack of guilt while lying (Brunell et al., 2011; Schröder-Abé and Fatfouta, 2019), consider lying as an appropriate communication strategy (Oliveira and Levine, 2008), and equally rate their own lie-telling and lie-detecting abilities higher than do non-narcissists (Giammarco et al., 2013; Jonason et al., 2014; Zvi and Elaad, 2018; Elaad, 2022).

However, there is a lack of studies using a more nuanced measure of lying when examining associations between deception and narcissism in romantic relationships. Regarding the dysfunctional relationship behaviors of agentic narcissists, the number of general lies or single scenarios do not provide much information about the different motives and reasons romantic partners lie to each other. We therefore propose a distinction between self-centered and other-oriented lies (see DePaulo and Kashy, 1998) to address the existing mixed results and thereby improve our understanding of narcissists’ lying behavior in romantic relationships. Theoretical support for the informative value of this distinction, especially when focusing on romantic relationships, is provided by several studies showing that other-oriented lies are told more often in romantic relationships compared to other relationship types (e.g., DePaulo and Kashy, 1998; Ennis et al., 2008). Thus, the specific motivation that drives lying in romantic relationships makes context-specific testing necessary, as established findings for predicting the frequency of lies are not necessarily transferable from other types of relationships (see also Cole, 2001).

By definition, self-centered lies are told to benefit oneself or to protect or enhance the liar’s psychological well-being/general interest; self-centered lies are also told to elicit a desired emotional response. In the context of romantic relationships, for example, the statement “I didn’t mind you picking up a girl last night” could represent a self-centered lie to the partner with the intention to appear untouchable. On the other hand, other-oriented lies are defined as lies told to benefit another person or to protect or enhance other persons’ psychological well-being/general interests. For example, claiming “I love the food you ordered for me” when this is in fact not true could represent an other-oriented lie in order not to make the partner feel bad (DePaulo and Kashy, 1998). It should be noted that within romantic relationships, lies that are actually told for the benefit of others also affect one’s own well-being, for instance, by helping to maintain the relationship or avoid an argument. In our study, we consider lies that are directly told to the romantic partner for self- and other-benefiting reasons; additionally, we also consider lies that relate to but are not directly directed to the romantic partner when such lies are told in the partner’s interest and therefore can be considered as other-oriented.

With respect to the general lying behavior of agentic narcissists, Jonason et al. (2014) found positive associations in a self-report study with the overall number of lies, number of self-gain lies (i.e., lies told to benefit oneself also known as self-centered lies), number of lies told for no reason, and their self-rated lying ability. The number of people lied to and the number of white lies (i.e., lies told to benefit another person also known as other-oriented lies) were not significantly related to agentic narcissism. Moreover, they found agentic narcissism to be positively associated with intersexual deception tactics of dominance and appearance. Consequently, based on the assumption that agentic narcissists consider lies as a legitimate communicative tool to achieve their self-motives, two things can be inferred. To begin with, in the context of romantic relationships, a somewhat increased use of other-oriented lies can be assumed, which can help protect the relationship. Nevertheless, agentic narcissists strive for dominance and self-enhancement, also in their romantic relationships, and thereby do not actually try to appear agreeable, suggesting a specifically strong positive connection with self-centered lying.

Because the examination of communal narcissism is still a niche in narcissism research, no findings are yet available on possible associations with attitudes toward lying or self-reported deceptive behavior. However, a survey of the existing evidence on communal narcissism and the lying behavior of agentic narcissists presents possible relations. It is important to emphasize that given the ambivalence of self-views of communal narcissists and conflicting corresponding perceptions by others (Nehrlich et al., 2019; Rogoza et al., 2023), studies based solely on self-reports must assume that communal self-enhancement leads to masking their actual deceptive behavior, at least to some degree. Nevertheless, regardless of whether it represents communal self-enhancement or actual behavior to appear more agreeable and altruistic, communal narcissists can be expected to more frequently report lying to their romantic partners than do non-narcissists because also here, deception can be used to strengthen the desired image of one’s own person as a romantic partner. The romantic partner could play a particularly important role here because if such a close person supports this communal image, it could in turn influence others’ views in the desired way. Considering further that communal narcissists are assumed to strategically use their prosociality to achieve self-motives by communal means (Giacomin and Jordan, 2015), especially a positive association with other-oriented lies can be assumed, representing a way to appear kinder and more sociable. However, since their own actions that do not correspond to a communal external image must sometimes be concealed in order to maintain it, self-centered lies can also be assumed to be told by communal narcissists.

### 1.1. Hypotheses

To gain a better understanding of the relations between the complementary facets of grandiose narcissism, which are both characterized by the desire to create a certain outward impression, the investigation of covert behaviors such as lying is particularly important. Based on our theoretical reasoning, we assume that agentic and communal narcissists partly use lies in different ways to achieve their self-motives of grandiosity, esteem, entitlement, and power (Gebauer et al., 2012) and therefore consider different types of lies (e.g., self-centered lies and other-oriented lies).

First, we predict that people higher in agentic and higher in communal narcissism will report increased deception toward their romantic partners (i.e., self-centered and other-oriented lies; Hypothesis 1). However, with a closer look at the reasons for what different types of lies are told, this hypothesis can be further specified.

Given the self-benefiting function of self-centered lies and given the assumption that agentic narcissists mainly strive to fulfill their motives of dominance and appearance within their romantic relationships (e.g., Campbell, 1999; Jonason et al., 2014; Zeigler-Hill et al., 2019), we predict agentic narcissism (compared with communal narcissism) will prove a stronger predictor for self-centered lies (Hypothesis 1a). One study conducted by Jonason et al. (2014) that showed agentic narcissism to significantly predict the self-reported number of self-centered lies, but not other-oriented lies, provides further support for this Hypothesis 1a.

Given the other-benefiting function of other-oriented lies and given the assumption that communal narcissists likewise use their romantic partners to fulfill their motive of self-enhancement through communal means, we predict that communal narcissism (compared with agentic narcissism) is a stronger predictor for other-oriented lies (Hypothesis 1b); past research revealing positive associations between the communal facet and agreeableness (Gebauer et al., 2012; Rogoza and Fatfouta, 2019) and self-reported altruism (Yang et al., 2018) provides further evidence for this Hypothesis 1b.

A third aim of the present study is to replicate the findings of Jonason et al. (2014), which is why we included their six-item questionnaire about general lying behavior. As mentioned, they found agentic narcissism to be positively related to the overall number of lies, number of self-gain lies, number of lies told for no reason, and their self-rated lying ability, but no significant association with the number of people lied to and the number of white lies. Jonason et al. (2014) were also interested in potential gender differences and found men (compared with women) to significantly report more overall lies and more lies told for no reason. Furthermore, men (compared with women) reported to have lied to more people, and they rated their lying ability better than women did.

## 2. Methods

We conducted two preregistered online studies for the investigation of our hypotheses. Both preregistration protocols are available on AsPredicted (Study 1^1^ and Study 2^2^), and Supplementary material is available on the Open Science framework (OSF).^3^ For both studies, relevant ethical guidelines were followed; we received ethical approval from the ethics committee of our university. Because both studies relied on similar procedures, we will report the methodology and results for both studies in a summarized form.

### 2.1. Subjects

We conducted an *a priori* power analysis using G*Power (Faul et al., 2007). With an assumed power of 0.90, setting Type I error rate at *p* < 0.05 and assuming an effect size of *r* = 0.20 (see Jonason et al., 2014), the power analysis for correlation (two-tailed) revealed a minimum sample size of *N* = 255.

Participants in both studies were recruited through private contacts, social media, and different survey platforms. They received no compensation and were informed that their participation was voluntary. We excluded individuals from both studies’ samples if they were under the age of 18, not in a romantic relationship, or had failed to complete the questionnaire.

Study 1 was conducted from May to July 2022. The final sample of Study 1 consisted of *N* = 298 participants (78.5% female, 19.5% male, 2.0% non-binary), with a mean age of 29.82 years (SD = 11.69). At 56%, most participants reported being student, followed by employed (31.5%), work-seeking (2.3%), being retired (3.0%), self-employed (2.0%), and other (5.0%). Study 2 was conducted from May to September 2022. The final sample of Study 2 consisted of *N* = 256 participants (75.0% female, 24.6% male, and 0.4% non-binary), with a mean age of 27.05 years (SD = 7.69). At 67.2%, again, most participants reported being student, followed by employed (27.7%), self-employed (1.6%), being retired (0.8%), and other (2.7%). All participants confirmed to currently be in a romantic relationship. In Study 1, 21.1% reported a relationship duration of less than 1 year, 24.5% of more than 1 year, 19.1% of more than 3 years, 19.8% of more than 5 years, and 15.4% of more than 10 years. In Study 2, 23.4% reported a relationship duration of less than 1 year, 25.8% of more than 1 year, 19.5% of more than 3 years, 22.3% of more than 5 years, and 9.0% of more than 10 years.

### 2.2. Procedure and measures

The study was conducted in the German language. First, participants confirmed participation requirements (i.e., the informed consent, currently in a romantic relationship). We then measured participants’ scores for agentic and communal narcissism, followed by questions concerning participants’ self-reported use of self-centered and other-oriented lies within their romantic relationship. We then presented the six items on self-reported deceptive behavior of Jonason et al. (2014). Participants then filled out demographic measures (i.e., age, gender, occupational status, ethnicity, and sexual preference) and were finally debriefed. All materials can be found in the Supplementary material (see section 3).

#### 2.2.1. Agentic narcissism

Agentic narcissism was assessed with the Narcissistic Personality Inventory (NPI; Raskin and Terry, 1988; German adaption by Schütz et al., 2004), which consists of 40 forced-choice dyads, including one narcissistic statement (e.g., “I like to be the center of attention”) and one non-narcissistic statement (e.g., “I prefer to blend in with the crowd”) each. The participants must choose the statement that best applies to them. The number of narcissistic statements selected by participants was then averaged to create an index of agentic narcissism.

#### 2.2.2. Communal narcissism

The Communal Narcissism Inventory (CNI; Gebauer et al., 2012) consists of 16 items that are answered on a scale ranging from 1 (*disagree strongly*) to 7 (*agree strongly*). All items were self-translated into German. Higher agreement with statements such as “I am generally the most understanding person” or “I am going to bring peace and justice to the world” indicate higher levels of communal narcissism. An index of communal narcissism was created by averaging all items.

#### 2.2.3. Types of lies

To measure different types of lies, we used an unpublished scale of Ennis et al. (2008). Originally, the items were created to measure deception regarding a close friend versus a stranger. For the purpose of our research question, we replaced the notations *close friend* and *stranger* by the notation *romantic partner*. In sum, the scale consisted of 12 items. Six items were used to measure the frequency of self-centered lies (i.e., lies that benefit oneself; e.g., “How often do you lie to your romantic partner in order to come out of situation looking the best?”), three items to measure the frequency of other-focused lies (i.e., lies that benefit the romantic partner; e.g., “How often do you lie to your romantic partner to prevent him/her from feeling hurt?”), and three items to measure the frequency of altruistic lies (e.g., “How often do you lie to others to protect your romantic partner from embarrassment?”). Responses were given on a scale ranging from 1 (*never*) to 7 (*very often*).

Because the distinction between other-focused lies and altruistic lies is unusual (see DePaulo and Kashy, 1998), we decided to summarize the three items to measure the frequency of other-focused lies with the three items to measure the frequency of altruistic lies to one dependent variable which we label as other-oriented lies. The six items to measure the frequency of self-centered lies were summarized to the second dependent variable. To confirm this two-factor solution, we conducted principal component analyses for both studies in which we indicated that only factors with eigenvalues ≥1 should be considered.

The Kaiser–Meyer–Olkin verified the sampling adequacy (Study 1: KMO = 0.88; Study 2: KMO = 0.90) and Bartlett’s test of sphericity was significant in both cases [Study 1: χ^2^(66) = 2,133.65, *p* < 0.001; Study 2: χ^2^(66) = 2,015.24, *p* < 0.001]. Thus, all requirements were fulfilled to proceed with the principal component analysis. In both cases, through the examination of Kaiser’s criteria and the scree-plot, empirical evidence supported the retention of two factors with eigenvalues greater than 1, which collectively explained 63.72% of the total variance in Study 1, and 66.22% in Study 2, respectively, with most items displaying the highest loading on the intended factor (see sections 1 and 2 in the Supplementary material).

#### 2.2.4. Replication

Besides our main analyses, we also aim to replicate the findings of Jonason et al. (2014). To do so, we asked participants (1) how many lies they told, (2) how many different people they lied to, (3) how many lies they told for their own benefit, (4) how many lies they told to avoid hurting another person, and (5) how many lies they told just because they felt like it. For each question, participants entered the estimated number of lies with respect to the past 7 days. Finally, we asked them (6) to assess how good they were at telling lies on a scale ranging from 1 (*very poor*) to 5 (*very good*). All items were self-translated into German.

## 3. Results

Table 1 shows descriptive statistics and zero-order correlation coefficients among all variables for both studies. Contrary to our predictions, there were no significant correlations between the two facets of grandiose narcissism and the two types of lies (all *p*s ≥ 0.117); the only exception was that agentic narcissism was positively related to other-oriented lies in Study 2 (*r* = 0.13, 95% CI [0.002, 0.24], *p* = 0.046).

**TABLE 1:** Descriptive statistics and Pearson’s correlations for study variables of studies 1 and 2.

Variables	Study 1			Study 2			(1)	(2)	(3)	(4)
	*M*	SD	α	*M*	SD	α				
1. Agentic narcissism	0.31	0.18	0.86	0.35	0.16	0.82	–	0.37*** [0.26, 0.47]	0.02 [−0.10, 0.14]	0.13* [0.002, 0.24]
2. Communal narcissism	4.06	0.85	0.88	4.13	0.93	0.91	0.43*** [0.33, 0.52]	–	0.04 [−0.08, 0.17]	0.10 [−0.03, 0.22]
3. Self-centered lies	2.47	1.13	0.88	2.47	1.21	0.90	0.08 [−0.03, 0.19]	−0.07 [−0.18, 0.05]	–	0.63*** [0.55, 0.70]
4. Other-oriented lies	3.35	1.28	0.87	3.46	1.30	0.88	−0.01 [−0.12, 0.11]	−0.06 [−0.18, 0.05]	0.54*** [0.46, 0.62]	–

Study 1: *N* = 298; Study 2: *N* = 256. Values below the diagonal refer to Study 1 and values above the diagonal refer to Study 2. Values in brackets are 95% confidence intervals. *p < 0.050; ***p < 0.001.

To further test our first hypothesis, we conducted four linear regression models. Two regression models each were run with self-centered lies as dependent variable and two regression models with other-oriented lies as the dependent variable. In every case, we added only one predictor per regression model (either agentic narcissism or communal narcissism; see Table 2). In both studies, neither agentic nor communal narcissism proved significant predictors of self-centered and other-oriented lies (all *p*s ≥ 0.117); the only exception was that the agentic facet of narcissism significantly positively predicted other-oriented lies in Study 2. However, the effect size must be interpreted as small, and the confidence interval is close to zero.

**TABLE 2:** Regression coefficients on self-centered and other-oriented lies of studies 1 and 2.

Study	DV	Predictor	*B*	SE *B*	95% CI		β	*p*
					LL	UL		
1	Self-centered lies	Agentic^a^	0.523	0.374	−0.21	1.26	0.01	0.162
	Self-centered lies	Communal^b^	−0.091	0.077	−0.24	0.06	−0.07	0.241
	Other-oriented lies	Agentic^c^	−0.044	0.426	−0.88	0.80	−0.01	0.918
	Other-oriented lies	Communal^d^	−0.096	0.088	−0.27	0.08	−0.06	0.276
2	Self-centered lies	Agentic^e^	0.166	0.475	−0.77	1.10	0.02	0.728
	Self-centered lies	Communal^f^	0.056	0.082	−0.11	0.22	0.04	0.492
	Other-oriented lies	Agentic^g^	1.017*	0.507	0.02	2.02	0.13	0.046
	Other-oriented lies	Communal^h^	0.138	0.087	−0.04	0.31	0.10	0.117

Study 1: *N* = 298; Study 2: *N* = 256. DV, dependent variable; 95% CI, confidence interval for B; LL, lower limit; UL, upper limit; Agentic, agentic narcissism; Communal, communal narcissism.

^a^*R*^2^ = 0.007; adj. *R*^2^ = 0.003; F(1,296) = 1.96, *p* = 0.162.

^b^*R*^2^ = 0.005; adj. *R*^2^ = 0.001; F(1,296) = 1.38, *p* = 0.241.

^c^*R*^2^ = 0.000; adj. *R*^2^ = −0.003; F(1,296) = 0.01, *p* = 0.918.

^d^*R*^2^ = 0.004; adj. *R*^2^ = 0.001; F(1,296) = 1.19, *p* = 0.276.

^e^*R*^2^ = 0.000; adj. *R*^2^ = −0.003; F(1,254) = 1.22, *p* = 0.728.

^f^*R*^2^ = 0.002; adj. *R*^2^ = −0.002; F(1,254) = 0.47, *p* = 0.492.

^g^*R*^2^ = 0.016; adj. *R*^2^ = 0.012; F(1,254) = 4.02, *p* = 0.046.

^h^*R*^2^ = 0.010; adj. *R*^2^ = 0.006; F(1,254) = 2.48, *p* = 0.117.

*p < 0.05.

To adequately test the predictive value of the two predictors agentic and communal narcissism against each other (as predicted in our Hypotheses 1a and 1b), we used the software *Psychometrica* (Lenhard and Lenhard, 2016). We performed comparisons between correlations from dependent samples. First, we compared the correlation between self-centered lies and communal narcissism (*r*_12_ = −0.07) with the correlation between self-centered lies and agentic narcissism (*r_13_* = 0.08) of Study 1; this analysis revealed a significant difference (*z* = −2.42, *p* = 0.008). Second, we compared the correlation between other-oriented lies and communal narcissism (*r*_12_ = −0.06) with the correlation between other-oriented lies and agentic narcissism (*r_13_* = −0.01) of Study 1; this analysis revealed a non-significant difference (*z* = −1.13, *p* = 0.130). Third, we compared the correlation between self-centered lies and communal narcissism (*r*_12_ = 0.04) with the correlation between self-centered lies and agentic narcissism (*r_13_* = 0.02) of Study 2; this analysis revealed a non-significant difference (*z* = 0.28, *p* = 0.388). Fourth, we compared the correlation between other-oriented lies and communal narcissism (*r*_12_ = 0.10) with the correlation between other-oriented lies and agentic narcissism (*r_13_* = 0.13) of Study 1; this analysis revealed a non-significant difference (*z* = −0.43, *p* = 0.334).

### 3.1. Replication

Following Jonason et al. (2014), we first calculated Pearson’s correlations between the six lie measures and the NPI scores representing the level of agentic narcissism. We further examined whether men and women differed in their lying behavior. Individuals who defined themselves as non-binary were therefore excluded from further analyses.

Of all six items, as reported in Table 3, only self-rated lying ability was positively correlated with agentic narcissism in Study 1 (*r* = 0.25, 95% CI [0.14, 0.35], *p* < 0.001) and Study 2 (*r* = 0.24, 95% CI [0.12, 0.35], *p* < 0.001). We further found the reported number of self-gain lies (i.e., self-centered lies) to be positively correlated with agentic narcissism in Study 1 (*r* = 0.13, 95% CI [0.10, 0.24], *p* = 0.033). Further, there were no significant gender differences in their self-reported deceptive behavior with the exception that in Study 2, men (*M* = 5.73, SD = 9.70) reported a higher number of lies compared to women [*M* = 3.35, SD = 5.73; *t*(253) = 2.73, *p* = 0.018].

**TABLE 3:** Pearson’s correlations for replication items with NPI and *t*-test results for sex differences of studies 1 and 2.

	Study 1								Study 2							
	*r*	95% CI	Men (*n* = 58)		Women (*n* = 234)		*t*(290)	*d*	*r*	95% CI	Men (*n* = 63)		Women (*n* = 192)		*t*(253)	*d*
			*M*	SD	*M*	SD					*M*	SD	*M*	SD		
Number of lies	0.10	[−0.01, 0.21]	7.95	16.64	5.30	13.06	1.30	0.19	0.02	[−0.11, 0.14]	5.73	9.70	3.35	5.73	2.37*	0.34
Number of people lied to	0.10	[−0.02, 0.21]	3.55	6.55	3.18	9.49	0.28	0.04	0.10	[−0.03, 0.22]	2.22	1.85	2.09	2.73	0.36	0.05
Number of self-gain lies	0.13*	[0.10, 0.24]	3.78	7.77	3.14	10.16	0.45	0.07	0.05	[−0.07, 0.17]	2.49	5.30	2.28	5.11	0.29	0.04
Number of white lies	0.11	[−0.01, 0.22]	3.07	8.46	3.00	10.21	0.50	0.01	0.10	[−0.03, 0.22]	2.95	5.72	2.00	4.91	1.28	0.19
Number of no reason for lies	0.10	[−0.02, 0.21]	0.72	2.04	0.78	6.59	−0.07	−0.01	0.06	[−0.06, 0.18]	0.71	1.62	1.11	6.90	−0.46	−0.07
Self-rated lying ability	0.25***	[0.14, 0.35]	3.24	1.16	2.93	1.31	1.65	0.24	0.24***	[0.12, 0.35]	3.17	1.21	2.99	1.36	0.94	0.14

We excluded participants who indicated non-binary as gender, therefore *N* = 292 for Study 1 and *N* = 255 for Study 2. 95% CI, 95% confidence interval for r. Note that all replication items referred to participants’ general lying behavior also apart from their romantic relationship. *p < 0.05; ***p < 0.001.

## 4. General discussion

We examined both facets of grandiose narcissism (i.e., agentic and communal narcissism; Gebauer et al., 2012) in relation to dishonesty in romantic relationships. We hypothesized that in this context, both agentic and communal narcissism would be positively related to the self-reported use of self-centered and other-oriented lies. We also predicted that agentic narcissism (compared with communal narcissism) would be a stronger predictor for self-centered lies, and communal narcissism (compared with agentic narcissism) would be a stronger predictor for other-oriented lies. However, the results of both studies revealed no reliable support for our hypotheses. It turned out that agentic and communal narcissism were unrelated to the self-reported use of self-centered and other-oriented lies toward participants’ romantic partners; the only exception emerged in Study 2, where agentic narcissism was positively correlated with other-oriented lies. The conducted regression analyses similarly revealed that only in Study 2 did agentic narcissism positively predicted other-oriented lies—and given that communal narcissism was totally unrelated to self-centered and other-oriented lies—our results weaken rather than strengthen confidence in our first hypothesis.

Consequently, our results contrast with previous studies reporting that agentic narcissists are more likely to lie to their partners in a hypothetical scenario (Azizli et al., 2016; Forsyth et al., 2021). Using the same scenario, however, Baughman et al. (2014) did not find agentic narcissists to be more prone to lying in romantic relationships. The distinction between self-centered and other-oriented lies in our study could not provide further clarification regarding these contradictions. In fact, the ambiguity becomes larger when comparing the results for the scales of Ennis et al. (2008) and the self-created questionnaire of Jonason et al. (2014) for measuring different types of lies. Using the scales of Ennis et al. (2008), results revealed (as mentioned above) a significant positive correlation between agentic narcissism and other-oriented lies (only in Study 2), but no significant correlation between agentic narcissism and self-centered lies. Using the items of Jonason et al. (2014), results revealed a significant positive association between agentic narcissism and self-centered lies (only in Study 1), but no significant correlation between agentic narcissism and other-oriented lies. At this point, however, it is important to point out the possibility that individuals with higher levels of agentic and communal narcissism could still be considered to lie more likely on a behavioral level (as already shown by some studies), however, maybe they are also more likely to conceal about their actual lying behavior, leading to the mixed empirical findings.

To adequately test our second and third hypotheses, we conducted comparisons between correlations from dependent samples for self-centered and other-oriented lies for both studies; we found that only in Study 1 the comparison between agentic and communal narcissism on self-centered lies revealed a significant difference. A closer look revealed that agentic narcissism was positively correlated with self-centered lies, but communal narcissism was negatively correlated. The finding that communal narcissists reported decreased self-centered lies contradicts our prediction; however, because this correlation must be interpreted as small and was not significant, we refrain from overinterpreting this result.

Overall, our results do not suggest individuals with higher scores in agentic narcissism to lie more often to their romantic partners than non-narcissistic individuals. Although agentic narcissism is commonly associated with poor relationship functioning, the mere frequency of lying on one’s own interest or on the interest of one’s partner does not seem to play a meaningful role in this regard. However, this does not preclude the possibility that agentic narcissism might be uniquely associated with deceptive behavior in romantic relationships (e.g., by lying more frequently in agentic domains; see Jonason et al., 2014). Since specifically for men the link between agentic narcissism and relationship satisfaction is mediated by the extent to which they seek to portray themselves as perfect to their partner (Casale et al., 2020), increased self-centered lying could also represent gender-specific behavior, which would have been more difficult to detect due to the relatively small proportion of men in our sample. However, from an investment model perspective (Campbell and Foster, 2002; Foster, 2008), in a situation where more frequent lying would be necessary to maintain the romantic partner’s admiration or to avoid conflicts (i.e., low satisfaction and high investment requirements), narcissists might be more prone to end the relationship and rather invest the effort in new acquaintances.

Regarding communal narcissism, our studies revealed no evidence for increased deception in romantic relationships, either in the interest of oneself or one’s partner. Although this refutes our initial assumptions, in retrospect, it could be a plausible outcome given the argument that communal narcissists could be expected to actively adjust their self-reported frequency downward to meet the common image that honesty is an important and respected norm. Correspondingly, self-reported lie frequency could be adjusted downward in the sense of communal self-enhancement and consequently to appear average. However, given the lack of further studies on the lying behavior of communal narcissists, this finding should not yet be given much weight. Therefore, the agency-communion model of narcissism should be given greater consideration in future deception research to adequately capture the entire construct of grandiose narcissism.

In addition to testing our main hypotheses, we aimed to replicate the findings of Jonason et al. (2014), using their self-report measures on general lying behavior. First, and consistent with the findings of Jonason et al. (2014), we found that agentic narcissism (measured with the NPI; Raskin and Terry, 1988) was positively related to self-rated lying ability when using their self-created scale. However, we could not replicate the findings that agentic narcissism is also positively related to the number of lies, number of self-gain lies (i.e., self-centered lies), and number of lies told for no reason. Regarding potential gender differences, and in line with Jonason et al. (2014), we only found men to report more overall lies compared with women. However, the original study also found men (compared with women) to report having lied to more people, having told more lies for no reason, and having an elevated lying ability; however, we did not find these gender differences in both of our studies. According to the current body of evidence, the association between agentic narcissism and a general tendency to lie is therefore in question. Although some studies (including ours) postulate for no significant association (e.g., Azizli et al., 2016), others postulate for an existing significant positive relationship (Zvi and Elaad, 2018; Forsyth et al., 2021). The same is true for the positive relationship between agentic narcissism and telling self-gain lies as postulated by Jonason et al. (2014): however, other works (including ours) were unable to replicate this despite using the same items (Zvi and Elaad, 2018). Lastly, the finding that agentic narcissists attribute a higher lying ability to themselves can be considered fairly robust (Jonason et al., 2014; Zvi and Elaad, 2018; Wissing and Reinhard, 2019; Elaad, 2022) and is coherent with regard to their self-enhancement tendencies in the agentic domain (Grijalva and Zhang, 2016).

It is important to note that both of our samples consisted solely of individuals in ongoing romantic relationships, which was not the case for studies that previously used the same six-item questionnaire of Jonason et al. (2014). Furthermore, our sample consisted mostly but not exclusively of students, as it was not the case in most previous studies. Hence, it is also possible that the social environment or age affect narcissists’ lying behavior in everyday life. For example, students versus professionals might have more casual acquaintances and be in an environment where lying is less likely to cause lasting harm. For future research, we therefore suggest examining these influences as moderating variables.

### 4.1. Limitations

We only used self-report measures in our studies, which did not allow us to draw reliable conclusions about participants’ actual deceptive behavior. Regarding potential associations between communal narcissism and deception, this seems to limit the informative value of our results, because communal narcissists in particular can be considered to show a discrepancy between their self-views and objective measures or peer reports. Therefore, future studies should extend the findings obtained using more behavioral measures of deception.

Further, recruiting the sample through private contacts, social media, and survey platforms carries the risk of biased sampling, which must be kept in mind when interpreting our results.

## 5. Conclusion

Summarized-as a take home message of this research-we again showed that people higher in agentic narcissism believe to be good liars, but this does not lead to higher self-reported frequencies of other-oriented and self-centered lies within participants actual romantic relationships. As a theoretical development compared to most other work in this field, we have been particularly interested in the relationship between communal narcissism and other-oriented lies, but also here no significant association appeared. Thus, narcissism promotes confidence in one’s own lying abilities, which are then, however, not exploited within the own romantic relationship.

## Data availability statement

Datasets and SPSS syntax of all studies can be found in online repositories. The name of the repository and accession link can be found in the article.

## Ethics statement

The studies involving human participants were reviewed and approved by the Ethics Committee of the University of Kassel. The patients/participants provided their written informed consent to participate in this study.

## Author contributions

NR, M-AR, and MM contributed to the conceptualization of the study. MM and NH collected the data. NH prepared a first draft. NR wrote sections of the manuscript. All authors involved in the statistical analyses, revised the manuscript, and approved the submitted version.

## Footnotes

1
https://aspredicted.org/m3zz2.pdf

2
https://aspredicted.org/z94ww.pdf

3
https://osf.io/pd24n/
