# Supplementary material for: Agentic and communal narcissism in predicting different types of lies in romantic relationships
Source: Front Psychol. 2023 Jul 3;14:1146732. doi: 10.3389/fpsyg.2023.1146732 (PMC10351034; doi:10.3389/fpsyg.2023.1146732)
Supplement: Supplementary file 1 [file Data_Sheet_1.PDF]

frontiers

Supplemental Material

Agentic and Communal Narcissism in Predicting Different Types of Lies in Romantic Relationships

Nico Harhoff, Nina Reinhardt*, Marc-André Reinhard, Michael Mayer

*** Correspondence:**
Corresponding Author
nina.reinhardt@uni-kassel.de

1 Principle Component Analysis for Study 1

Results From the Rotated Component Matrix for Study 1

Item	Factor Loadings	
	Factor 1	Factor 2
Factor 1: Self-centered lies		
1. How often do you lie to your partner to protect yourself from embarrassment and/or shame?	.81	
2. How often do you lie to your partner to get information for your own benefit?	.79	
3. How often do you lie to your partner to prevent him/her from knowing things about you that you don't want him/her to know?	.76	.29
4. How often do you lie to your partner to get out of a situation where you have to do something you are not interested in?	.74	.23
5. How often do you lie to your partner to get out of a situation and look your best?	.74	.20
6. How often do you lie to your partner to cover up something you did that was wrong?	.73	.24
Factor 2: Other-oriented lies		
7. How often do you lie to your partner to make him/her feel better?		.85
8. How often do you lie to your partner to protect him/her from feeling hurt?	.18	.78
9. How often do you lie to your partner to protect him/her from being disappointed?		.77
10. How often do you lie to protect the secrets your partner has shared with you in confidence?	.48	.68
11. How often do you lie so that your partner is seen in a more positive light by others?	.45	.67
12. How often do you lie to others to protect your partner from embarrassment?	.46	.64

Note. Study 1: $N = 298$.

2 Principle Component Analysis for Study 2

Results From the Rotated Component Matrix for Study 2

Item	Factor Loadings	
	Factor 1	Factor 2
Factor 1: Self-centered lies		
1. How often do you lie to your partner to protect yourself from embarrassment and/or shame?	.82	.12
2. How often do you lie to your partner to get information for your own benefit?	.81	.15
3. How often do you lie to your partner to prevent him/her from knowing things about you that you don't want him/her to know?	.78	.23
4. How often do you lie to your partner to get out of a situation where you have to do something you are not interested in?	.78	.13
5. How often do you lie to your partner to get out of a situation and look your best?	.74	.20
6. How often do you lie to your partner to cover up something you did that was wrong?	.72	.30
Factor 2: Other-oriented lies		
7. How often do you lie to your partner to make him/her feel better?	.62	.57
8. How often do you lie to your partner to protect him/her from feeling hurt?	.56	.54
9. How often do you lie to your partner to protect him/her from being disappointed?	.22	.84
10. How often do you lie to protect the secrets your partner has shared with you in confidence?		.82
11. How often do you lie so that your partner is seen in a more positive light by others?	.30	.75
12. How often do you lie to others to protect your partner from embarrassment?	.58	.59

Note. N = 256.

3 Materials of Studies 1 and 2 (identical)

Filter item

"I hereby certify that I am at least 18 years old and currently in a romantic relationship."

Response options:

- *Yes, I am at least 18 years old and currently in a romantic relationship.*
- *No (exclusion)*

Narcissistic Personality Inventory (NPI)

On the following two pages you will be presented with a total of 40 different paired statements.

For each paired statement, please select the one with which you identify most.

If you identify with both equally, please select the one that is most important to you.

A. I have a natural talent for influencing people.	B. I am not good at influencing people
A. Modesty doesn't become me.	B. I am essentially a modest person.

A. I would do almost anything on a dare.	B. I tend to be a fairly cautious person.
A. When people compliment me I sometimes get embarrassed.	B. I know that I am good because everybody keeps telling me so.
A. The thought of ruling the world frightens the hell out of me.	B. If I ruled the world it would be a better place.
A. I can usually talk my way out of anything.	B. I try to accept the consequences of my behavior.
A. I prefer to blend in with the crowd.	B. I like to be the center of attention.
A. I will be a success.	B. I am not too concerned about success.
A. I am no better or worse than most people.	B. I think I am a special person.
A. I am not sure if I would make a good leader.	B. I see myself as a good leader.
A. I am assertive.	B. I wish I were more assertive.
A. I like to have authority over other people.	B. I don't mind following orders.
A. I find it easy to manipulate people.	B. I don't like it when I find myself manipulating people.
A. I insist upon getting the respect that is due me.	B. I usually get the respect that I deserve.
A. I don't particularly like to show off my body.	B. I like to show off my body.
A. I can read people like a book.	B. People are sometimes hard to understand.

A. If I feel competent I am willing to take responsibility for making decisions.	B. I like to take responsibility for making decisions.
A. I just want to be reasonably happy.	B. I want to amount to something in the eyes of the world.
A. My body is nothing special.	B. I like to look at my body.
A. I try not to be a show off.	B. I will usually show off if I get the chance.
A. I always know what I am doing.	B. Sometimes I am not sure of what I am doing.
A. I sometimes depend on people to get things done.	B. I rarely depend on anyone else to get things done.
A. Sometimes I tell good stories.	B. Everybody likes to hear my stories.
A. I expect a great deal from other people.	B. I like to do things for other people.
A. I will never be satisfied until I get all that I deserve.	B. I take my satisfactions as they come.
A. Compliments embarrass me.	B. I like to be complimented.
A. I have a strong will to power.	B. Power for its own sake doesn't interest me.
A. I don't care about new fads and fashions.	B. I like to start new fads and fashions.
A. I like to look at myself in the mirror.	B. I am not particularly interested in looking at myself in the mirror.
A. I really like to be the center of attention.	B. It makes me uncomfortable to be the center of attention.

A. I can live my life in any way I want to.	B. People can't always live their lives in terms of what they want.
A. Being an authority doesn't mean that much to me.	B. People always seem to recognize my authority.
A. I would prefer to be a leader.	B. It makes little difference to me whether I am a leader or not.
A. I am going to be a great person.	B. I hope I am going to be successful.
A. People sometimes believe what I tell them.	B. I can make anybody believe anything I want them to.
A. I am a born leader.	B. Leadership is a quality that takes a long time to develop.
A. I wish somebody would someday write my biography.	B. I don't like people to pry into my life for any reason.
A. I get upset when people don't notice how I look when I go out in public.	B. I don't mind blending into the crowd when I go out in public.
A. I am more capable than other people.	B. There is a lot that I can learn from other people.
A. I am much like everybody else.	B. I am an extraordinary person.

Response options:

- *either A or B*

Communal Narcissism Inventory (CNI)

People have all kinds of private thoughts about themselves. From person to person, these self-thoughts can vary quite a lot in content.

We are interested in the sort of self-thoughts you possess. Below you will find a list of self-thoughts you may have.

For each self-thought, please indicate whether you have this or a similar thought.

Be as honest as possible. Remember, your responses are totally anonymous.

I am the most helpful person I know.
I am going to bring peace and justice to the world.
I am the best friend someone can have.
I will be well known for the good deeds I will have done.
I am (going to be) the best parent on this planet.
I am the most caring person in my social surrounding.
In the future I will be well known for solving the world's problems.
I greatly enrich others' lives.
I will bring freedom to the people.

I am an amazing listener.

I will be able to solve world poverty.

I have a very positive influence on others.

I am generally the most understanding person.

I'll make the world a much more beautiful place.

I am extraordinarily trustworthy.

I will be famous for increasing people's well-being.

Response options:

- *Scale ranging from 1 (disagree strongly) to 7 (agree strongly)*

Self-centered lies

Lying in romantic relationships is a natural phenomenon that happens in every relationship and is completely normal! In the following, we will show you possible reasons that can lead to deceptive behavior in romantic relationships.

How often do you lie to your partner to protect yourself from embarrassment and/or shame?

How often do you lie to your partner to get information for your own benefit?

How often do you lie to your partner to prevent him/her from knowing things about you that you don't want him/her to know?

How often do you lie to your partner to get out of a situation where you have to do something you are not interested in?

How often do you lie to your partner to get out of a situation and look your best?

How often do you lie to your partner to cover up something you did that was wrong?

Response options:

- *Scale ranging from 1 (never) to 5 (very often)*

Other-oriented lies

Again, we will show you possible reasons that can lead to deceptive behavior in romantic relationships.

How often do you lie to your partner to make him/her feel better?

How often do you lie to your partner to protect him/her from feeling hurt?

How often do you lie to your partner to protect him/her from being disappointed?

How often do you lie to protect the secrets your partner has shared with you in confidence?

How often do you lie so that your partner is seen in a more positive light by others?

How often do you lie to others to protect your partner from embarrassment?

Response options:

- *Scale ranging from 1 (disagree strongly) to 7 (agree strongly)*

Replication items (Jonason et al., 2014)

Please think about the period of the past 7 days when answering the following 5 questions.

Please try to remember as best you can!

How many lies have you told?

How many different people have you lied to?

How many lies have you told for your own self-gain?

How many lies have you told in order not to hurt another person?

How many lies have you told just because you felt like it?

Text field (number of lies)

How good do you think you are at lying?

Response options:

- *Scale ranging from 1 (very poor) to 5 (very good)*

Demographics

Finally, please provide some demographic information!

How old are you?

Text field (age)

Which gender do you identify with?

Response options:

- *(1) male*
- *(2) female*
- *(3) divers*

What is your sexual preference?

Response options:

- *(1) male*
- *(2) female*
- *(3) both*
- *(4) other*
- *(5) I do not want to specify*

How long have you been in your current relationship?

Response options:

- *(1) less than 1 year*
- *(2) more than 1 year*
- *(3) more than 3 years*
- *(4) more than 5 years*
- *(5) more than 10 years*

What is your current employment status?

Response options:

- *(1) employee*
- *(2) self-employed*
- *(3) student*
- *(4) jobseeker*
- *(5) pensioner*

What is your highest educational qualification?

Response options:

- *(1) no school diploma*
- *(2) elementary/middle school diploma (Hauptschulabschluss)*
- *(3) middle school diploma (Mittlere Reife)*
- *(4) high school diploma (Abitur)*
- *(5) completed vocational training*
- *(6) University of Applied Sciences degree*
- *(7) University (Bachelor's degree)*
- *(8) University (Master's degree)*
- *(9) University (Doctoral degree)*
- *(10) other*
